# Factors shaping how clinical educators use their educational knowledge and skills in the clinical workplace: a qualitative study

**DOI:** 10.1186/s12909-016-0590-8

**Published:** 2016-02-18

**Authors:** Koshila Kumar, Jennene Greenhill

**Affiliations:** Flinders University Rural Clinical School, Flinders University, GPO Box 2001, Adelaide, 5001 South Australia Australia

**Keywords:** Clinical education, Educationally-focussed professional development, Clinical educators, Workplace affordances

## Abstract

**Background:**

In order to consolidate their educational knowledge and skills and develop their educational role, many clinicians undertake professional development in clinical education and supervision. It is well established that these educationally-focussed professional development activities have a positive impact. However, it is less clear what factors within the clinical workplace can shape how health professionals may use and apply their educational knowledge and skills and undertake their educational role. Looking through the lens of workplace affordances, this paper draws attention to the contextual, personal and interactional factors that impact on how clinical educators integrate their educational knowledge and skills into the practice setting, and undertake their educational role.

**Methods:**

Data were gathered via a survey of 387 clinical educators and semi-structured interviews with 12 clinical educators and 6 workplace managers. In this paper, we focus on analysing and reporting the qualitative data gathered in this study. This qualitative data were subject to a thematic analysis and guided by theoretical constructs related to workplace affordances.

**Results:**

Three key themes were identified including contextual, personal and interactional factors. Contextual elements referred to organisational structures and systems that impact on participants’ educational role, how participants’ clinical education role was articulated and configured within the organisation, and how the organisation shaped the educational opportunities available to clinicians. Personal factors encompassed clinicians’ personal motivations and goals to teach and be involved in education, develop their own educational skills and function as a role model for students. Interactional factors referred to the professional interactions and networks through which clinicians shared their educational knowledge and skills and further consolidated their profile as educational advocates in their workplace.

**Conclusions:**

There are a number of contextual, personal and interactional factors which interrelate in complex ways and impact on how clinical educators use their educational knowledge and skills and undertake their educational role in the clinical setting. To fully realise the potential of and fulfil the requirements of their educational role, clinical educators need to be provided appropriate experiential and meaningful workplace opportunities and the guidance to use, share and reflect on their educational knowledge and skills in the clinical workplace.

## Background

Many clinicians undertake educationally-focused professional development to develop and consolidate their educational knowledge and skills and their educational role. It is well established that such programs and activities work [1–4]. A recent systematic review showed that educationally-focussed professional development activities within health are associated with positive effects, mostly in relation to participant satisfaction and changes in attitudes, knowledge and skills and behaviour [5]. Positive outcomes have also been observed at the organisational level [6, 7]. However, this focus on outcomes has distracted from a more holistic and theoretically-informed consideration of how the broader organisational context within which healthcare professionals are located shapes what they do with their educationally-focussed knowledge and skills. As a result, there is an increasing call for theoretically framed [5] studies that illustrate how clinicians integrate their educational knowledge and skills into the practice setting [8].

Workplace affordances as a theoretical framework, was originally proposed by Stephen Billett in the context of student learning in work-based settings [9]. This framework attends to the contextual elements (e.g. workplace environments, structures and systems) and personal factors (e.g. individual agency and engagement) [9] which can shape learning within practice settings. Workplace affordances has recently been utilised as a conceptual lens to explore how other groups such as medical educators may use and integrate new learning [10]. For example, Onyura and colleagues showed that affordances can be conceptualised on a gradient from facilitating, constraining to inhibiting, where the former refers to aspects that support the application of empirical knowledge while the latter refers to elements that partially or entirely limit an individual’s capacity to integrate empirical knowledge into their practice [10]. In this paper, we use workplace affordances as a theoretical lens to elucidate the factors which shape how clinical educators working in the health service may integrate the knowledge and skills into the clinical setting. To expand on the original framework of workplace affordances, there is also potential to consider the role of interactional elements such as social and professional relationships and networks.

The aim of this paper is to explore and illustrate the factors that shape how clinical educators use and apply their educational knowledge and skills in the clinical workplace and undertake their educational role.

## Methods

This study was approved by the Flinders University Social and Behavioural Research Ethics Committee (project number: 6364) and the SA Health Human Research Ethics Committee (project number: HREC/13/SAH/144) and associated Local Health Networks.

### Participants

This study was conducted between 2013 and 2014 and involved a cross sectional sample of 387 clinical educators in South Australia. The majority of these clinical educators were: from a nursing background, employed in the public health system and in the acute care/hospital setting, and working in a metropolitan location (see Table 1). Of these, twenty-five clinical educators and their workplace managers were purposively approached for participation in follow up data collection with reference to demographic variables of interest including profession, sector of employment, organisation and location of work. Twelve clinical educators and six workplace managers agreed to participate in the follow up data collection.

**Table 1:** Clinical educator demographic information

	Frequency	Percentage
Profession		
Allied health	88	22.7 %
Medicine	27	7 %
Midwifery	21	5.4 %
Nursing	195	50.4 %
Other	4	1 %
Not answered	52	13.4 %
Area of practice		
Primary care	59	15.2 %
Aged care	56	14.5 %
Acute care	107	27.6 %
Mental health	14	3.6 %
Rural health	101	26.1 %
Other	2	0.5 %
Not answered	48	12.4 %
Organisation where employed		
Public	225	58.1 %
Private	47	12.1 %
Not for profit	11	2.8 %
Not answered	104	26.9 %
Location where employed		
Rural	101	26.1 %
Metropolitan	180	46.5 %

### Data collection

Both quantitative and qualitative data was collected in this study, but this paper specifically focuses on reporting the qualitative component. The first source of data for the study was a survey which was administered in both online and paper-based modes. This survey collected demographic information, quantitative data related to participants’ self-rated knowledge, skill, awareness, confidence and willingness in relation to clinical education, and qualitative data about participants’ educational role, how they used educational knowledge and skills in the clinical workplace and their supervision experiences. The latter open-ended questions were a specific source data for this paper.

A second source of qualitative data for this paper was the semi-structured interviews which explored in-depth clinical educators’ and workplace managers’ views regarding the use of educational knowledge and skills within the clinical practice setting and factors which impact on how healthcare professionals undertake their educational role. Prior to each interview, all participants submitted a signed written consent form via email. All interviews were conducted over the phone by a research assistant not known to the participants, were transcribed verbatim, and de-identified by removing any identifying information such as names of people and organisations prior to analysis and reporting. Interviews ranged from 15 to 34 min.

### Data analysis

In this paper, we focus specifically on analysing and reporting the qualitative data collected in this study relating specifically to how health professionals use their educational knowledge and skills in the clinical workplace and undertake their educational role. As described, this qualitative data encompassed both open-ended survey responses and interview data. This data were entered into NVivo and a thematic analysis [11] was undertaken. The analytical process involved both authors reading interview transcripts and the open-ended survey responses to familiarise themselves with the qualitative data. Next, open coding was undertaken (without reference to the theoretical lens) on a proportion of the transcripts to generate multiple codes which illustrated the key experiences described by respondents. Then, connections among open codes were discussed by both authors and organised into themes. Next, these themes were further reviewed and refined by one author (KK) to produce a thematic map for the entire dataset. The final step involved a deductive analysis [12] informed by the constructs articulated within workplace affordances [9] which was used as a sensitising and organising theoretical framework.

## Results

There were several interrelated contextual, personal and interactional factors impacting on how health professionals used and integrated their educational knowledge and skills into the clinical setting and undertook their educational role. These factors are described in more detail with reference to participant quotes which are labelled according to participant type (clinical educator or workplace manager) and where they are sourced from (survey or interview and number).

### Contextual factors

Contextual factors refer to workplace systems and frameworks that impact on participants’ educational role, and also to how health professionals’ education role was articulated and configured, and recognised within their organisation. In relation to the former, workplace managers noted that clinical education was often driven by workplace accreditation or quality assurance requirements. For example, some managers noted that a recently introduced Clinical Governance Framework which outlined a new model of staff supervision “*dovetailed*” with the focus on clinical education. This governance framework functioned to normalise education within the clinical practice environment and provide a strong rationale or the time, energy and resources that needed to be invested in supporting and developing staff their educational roles.

“*We had some rules around what we had to do around clinical supervision and then we had some ability to support people to do that. To feel confident that it was the right thing to do, so instead of feeling pressured, no I've got too much work to do, I can't dedicate that time. Suddenly it became - well not suddenly but it became the normal that this is what's acceptable and this is what is an appropriate level of support to provide to your staff*.” (Manager/interview INT016)

Clinical educators noted that high quality clinical supervision was highly dependent on being able to spend adequate time with students as part of the usual workplace routine. However, clinical service provision was an over-riding priority and there was often a need to manage a large clinical workload. Together, this made it difficult for clinicians to appropriately balance their service and education roles and responsibilities.

*“The time it takes to do good supervision, and even though that pays off, is difficult to balance with a demanding clinical load”.* (Clinical supervisor/ interview INT01)

It was clear that healthcare professionals’ educational role could be articulated in different ways within the clinical workplace. Some participants’ educational role was formally configured and articulated ‘on paper’ e.g. in their position title. Participants who had a formal student or staff education component to their role actively participated in a range of activities such as: direct student supervision; developing and delivering staff orientation; supporting transition to practice for new or junior staff; facilitating small group learning; and planning and developing educational resources and tools for use in their local setting. These participants’ educational profile was widely recognised within their organisation including by peers, students and managers in the clinical workplace. They were often sought out for their educational expertise and some of them took on a role as educational advisors in helping others negotiate supervision expectations, sharing information about education-related professional development resources and opportunities and providing informal education-focused career counselling. Workplace managers noted that individuals with a formal educational role and profile tended to do ‘*a lot with our other staff and provide them with some tactics and skills around supervision’.*

*“It also gives me the ability to help out when there's issues and to sort of be a buffer zone with knowledge about students and their expectations”.* (Clinical supervisor/survey)

In contrast, other participants in this study did not have a formal student teaching or staff development role and thus had a limited educational profile within the organisation. These individuals reported having variable engagement and no longitudinal involvement in educational activities and events in the workplace. They tended to adopt relatively informal and ad-hoc approach to using or communicating their educational knowledge and skills within the clinical setting and were not as commonly recognised or sought out for their expertise as their peers were.

*“Probably, without making a real conscious effort, in discussions and that, I probably have used it, without really realising it”.* (Clinical supervisor/survey)

Some of these participants reported they were able to incorporate their educational knowledge and skills ‘*in a less formal manner’* into their every-day clinical interactions particularly within the multidisciplinary team context. Although this was perceived by some workplace managers as an appropriate way for those who had no formal involvement in student or staff education to use their educational knowledge and skills, it is questionable whether this was seen as a meaningful activity by the healthcare professionals themselves.

*“It's about how people utilise those skills perhaps in a less formal manner. It's about how they integrate that into their day to day work, when they're working within the multidisciplinary team”.* (Manager/interview INT017)

In summary, this theme revealed that there were a range of workplace factors that can impact on how participants used their educational knowledge and skills in the practice setting and undertook their educational role. In particular, the manner in which healthcare professionals’ roles were formally and informally configured and recognised within their workplace shaped the nature and extent of their access to and engagement in various educational events and activities, as well as the opportunities they had to put their educational knowledge and skills into action.

### Personal factors

Personal factors refer specifically to clinicians’ personal motivations, self-efficacy beliefs, and expectations in relation to their role as an educator. Participants expressed strong personal beliefs about the importance of providing students with hands-on opportunities for learning through clinical service provision. Some viewed that a core part of their role was to encourage students to venture out of their comfort zone and take ownership of and responsibility for their learning.

“*It’s about getting the student to take responsibility for themselves, their own learning and to become involved and actually get those hands-on skills that they need to do during placement. It's like I said, if you don't push them, well, not so much push them, but encourage them I suppose to be hands-on, they don't get it”*. (Clinical supervisor/ interview INT04)

Others viewed their role more broadly in terms of helping students develop an enhanced sense of fit and belonging within the clinical environment and negotiate and construct their professional identity as a health professional.

“*Making sure the students knew exactly where they were at, letting them know that they've got the autonomy to let the staff know what they can and cannot do*”. (Clinical supervisor/ interview INT010)

Participants recognised their powerful position as a role model, both in relation to clinical and non-clinical or professionalism skills, and this intersected to further shape their sense of self-efficacy as a clinical educator.

“*I think I'm more aware of my own interactions with the clients and what I'm doing to make sure that it is the right way so they actually get the right impression. So basically I'm a good role model and hopefully teaching them good habits*”. (Clinical supervisor/survey)

Workplace managers also noted that health professionals also consistently reflected on the range of higher level competencies and capabilities associated with their educational role, and this was often a catalyst for integrating their educational knowledge and skills into practice.

‘*it has given them a more holistic view of what their role is, it's about much more than just teaching someone to be a nurse’* (Manager/interview INT03)

Overall, this theme illustrated a range of personal factors including healthcare professionals’ personal beliefs, values and motivations in relation to student or staff education and perceptions of their own role as educational advocates and role models in the clinical environment, impacted on how they developed a more holistic view of their own role and integrated their educational knowledge and skills into the clinical practice environment.

### Interactional factors

Interactional factors refer to the social and professional interactions and relationships through which clinical educators sought to share and consolidate their educational knowledge and skills, and leverage their educational role. Participants reported drawing on their professional and workplace networks and connections to organise a range of learning activities which provided students with the opportunity to hone not just their clinical skills but also their communication and interpersonal skills.

“*I actually also got them involved in the full gamut of aged care and also helped work on their communication skills for the elderly. I made sure they had a lot of contact that wasn't necessarily just clinically based but learn to build rapport with residents*”. (Clinical supervisor/interview INT01)

It was clear that other participants were able to leverage their professional networks to create positive change particularly in workplaces where there was some resistance towards clinical education. Some participants reported using their knowledge and skills to try and transform staff attitudes towards education. For example, one clinical educator spoke about the need to ‘*stand up to other staff who get frustrated and tell them that the student is learning and they need to be patient’*.

“*I try and change their [staff] way of thinking, like how to be helpful to students, to be a good role model on the ward level, because there is always people who don't want to have anything to do with students and don't think it is part of their role to teach or influence students.*” (Clinical supervisor/interview INT05)

Workplace managers also corroborated these findings by reporting that some participants took on an educational advocacy role in the clinical workplace and were involved in ‘*advocating on behalf of what the students actually require in order to get a good learning experience*’. They also noted some were able to extend or challenge current educational thinking and practice within the organisation in terms of ‘*working with students in ways not thought of before*’.

## Discussion

This study explored the factors shaping how healthcare professionals use their educational knowledge and skills and undertake their educational role within the clinical workplace, as perceived by clinical educators and workplace managers. Workplace affordances [9] was used as a theoretical lens to provide insight into several interrelated factors which could enable or impede how participants put their knowledge and skills into action. Contextual factors included workplace structures and systems, how participants’ educational roles were formally or informally configured, and the visibility of their education profile within the organisation. Personal factors related to participants’ personal goals, motivations, self-efficacy beliefs and their level of agency i.e. the extent to which they were proactively shaping their own educational role and profile within the clinical setting. Interactional factors referred to the engagement between clinical educators and others in their organisation and the social and professional relationships that underpin what is intrinsically an interactive and dynamic process. Overall, our findings point to the complex and interrelated factors impacting on healthcare professionals as they navigate between their clinical and education roles and responsibilities within the health service. Clinicians’ level of personal engagement and investment in education and how this is secured and sustained has been identified as a key influencing mechanism in the context of faculty development [13]. In particular, social and professional relationships within the clinical workplace can form the basis of networks of practice [14] within which educators can consolidate and leverage their educational role.

A key underpinning finding in this study is that participants were developing enhanced understandings of their educational role and responsibilities within the health practice setting and constructing a more nuanced professional identity. Professional identity within health is primarily defined and constructed in relation to the primary tenet of and core responsibilities associated with clinical service provision, rather than education. However, it has previously been identified that being and becoming a clinical educator involves not just the acquisition of knowledge and skills, but also the development of a sense of self commensurate with one’s educational role and responsibilities [15]. Our findings are consistent with the literature which shows that experimenting with new identities is one of the main ways in which individuals develop holistic understandings and views of their role [16]. Health professional educators need to be engaged in ongoing negotiation and development to balance and extend their existing professional (i.e. clinical) identity to accommodate their emerging educational identity and role [17].

### Implications

There are several implications emerging from this study about what can be done to facilitate and sustain how healthcare professionals use and apply their educational knowledge and skills within the health service, and to help nurture their emerging educational identity. First, clinicians need to be appropriately supported in developing, maintaining and enhancing their formal and informal educational profile and pursuing their educational interests in the clinical workplace. This can be achieved by providing individuals with the opportunity to participate in a range of meaningful, authentic and practice-based activities (outside of specific professional development events) related to education. These can include but are not limited to: providing direct student or staff supervision; planning, developing or evaluating curriculum and educational resources; reporting on critical appraisals of the educational literature and new innovations or theories; engaging in peer review related to curriculum design or delivery; conducting local educational evaluation or research; or providing informal career counselling in relation to educationally-focussed professional development. Participation in such activities will help healthcare professionals use, share, and critically reflect on their educational knowledge and skills and their role and responsibilities in relation to student and/or staff education. Such activities will also be important to facilitate connectivity, dialogue and shared learning across various social and professional networks in the workplace including across different health professions. Overall, these activities can function to socialise individuals into the expectations, functions and norms of their emerging educational identity [17].

### Strengths and limitations

This study is limited in that it relies on self-reported data from clinical educators in one state in Australia. However, the strength of findings is enhanced by the: cross sectional approach to surveying a large cohort of clinical educators; triangulation of data across survey and interview methods and participant types (i.e. clinical educators and workplace managers); and use of an established theoretical framework.

## Conclusions

This study has shown there are a number of contextual, personal and interactional factors which intersect and interact to shape how clinical educators use and integrate their educational knowledge and skills into the clinical practice setting and undertake their educational role within the health service. Clinical educators need to be afforded relevant, authentic and experiential workplace opportunities to use, share and critically reflect on their educational knowledge, skills and role in order to develop a more nuanced professional identity in relation to education.
